# COVID-19 in Joint Ageing and Osteoarthritis: Current Status and Perspectives

**DOI:** 10.3390/ijms23020720

**Published:** 2022-01-10

**Authors:** Marianne Lauwers, Manting Au, Shuofeng Yuan, Chunyi Wen

**Affiliations:** 1 Department of Biomedical Engineering, The Hong Kong Polytechnic University, 11 Yuk Choi Rd, Hung Hom, Hong Kong; mmlauwe@polyu.edu.hk (M.L.); manting.au@polyu.edu.hk (M.A.); 2 Department of Microbiology, The University of Hong Kong, Pok Fu Lam, Hong Kong; yuansf@hku.hk

**Keywords:** long-COVID, osteoarthritis, musculoskeletal aging

## Abstract

COVID-19 is a trending topic worldwide due to its immense impact on society. Recent trends have shifted from acute effects towards the long-term morbidity of COVID-19. In this review, we hypothesize that SARS-CoV-2 contributes to age-related perturbations in endothelial and adipose tissue, which are known to characterize the early aging process. This would explain the long-lasting symptoms of SARS-CoV-2 as the result of an accelerated aging process. Connective tissues such as adipose tissue and musculoskeletal tissue are the primary sites of aging. Therefore, current literature was analyzed focusing on the musculoskeletal symptoms in COVID-19 patients. Hypovitaminosis D, increased fragility, and calcium deficiency point towards bone aging, while joint and muscle pain are typical for joint and muscle aging, respectively. These characteristics could be classified as early osteoarthritis-like phenotype. Exploration of the impact of SARS-CoV-2 and osteoarthritis on endothelial and adipose tissue, as well as neuronal function, showed similar perturbations. At a molecular level, this could be attributed to the angiotensin-converting enzyme 2 expression, renin-angiotensin system dysfunction, and inflammation. Finally, the influence of the nicotinic cholinergic system is being evaluated as a new treatment strategy. This is combined with the current knowledge of musculoskeletal aging to pave the road towards the treatment of long-term COVID-19.

## 1. Introduction

### 1.1. Acute COVID-19 Infection

Since the discovery of the first human coronavirus in 1965, different coronaviruses evolved, causing mostly respiratory problems that resemble a common cold in infected individuals. The most well-known human coronaviruses are SARS-CoV (severe acute respiratory syndrome coronavirus), MERS-CoV (middle east respiratory syndrome coronavirus), and more recently SARS-CoV-2 (severe acute respiratory syndrome-coronavirus-2) [1,2]. The latter, which is the virus responsible for COVID-19 infections, spreads quickly over the world. The most vulnerable populations are the elderly and individuals with chronic diseases [3]. Up until December 2021, 5.3 million deaths were recorded worldwide by WHO. SARS-CoV-2 is a single positive-strand RNA virus consisting of 4 structural proteins: N(nucleocapsid), S(spike), M(membrane) and E(envelope). The N-protein forms a helical capsid to protect the genome, the M and E protein form the viral structure, and the S protein is responsible for viral entry into the host cell. The S glycoprotein is cleaved by the host proteinase into S1 and S2. S1 binds to the host receptor, angiotensin-converting enzyme 2 (ACE2), while S2 is responsible for the fusion between the host and viral membranes [2,4]. The most common route of transmission is exposure to infectious respiratory fluids carrying the coronavirus. A SARS-CoV-2 infection is characterized by non-specific respiratory and constitutional symptoms such as cough, fever, diarrhea, and fatigue. However, a large percentage of the infected population is asymptomatic. The most severe cases cumulate into severe pneumonia, respiratory failure, organ damage, and dysfunction, and eventually death [1,2].

### 1.2. Post-Acute and Chronic COVID-19

A SARS-CoV-2 infection is mostly known to be an acute infection manifesting 4–5 days after exposure and lasting for 7–10 days. However, 87% of the hospitalized patients and 35% of the out-of-hospital patients have persistent symptoms continuing for several weeks or months, irrespective of their viral status. Depending on the duration of the symptoms, SARS-CoV-2 infections can cause post-acute COVID when they last longer than 3 weeks but shorter than 12 weeks, while long term effects of COVID, or long-COVID, last longer than 12 weeks [5]. Most long-COVID patients no longer have a viral load and are thus microbiologically healthy despite suffering from persistent symptoms. Long-COVID symptoms vary between individuals and include fatigue, cough, shortness of breath, dyspnea, joint pain, loss of smell and taste, attention disorder, hair loss, and headache [6,7,8]. Long-COVID can be classified into different phenotypes according to the most prominent residual symptoms such as: cardio-respiratory phenotype, fatigue phenotype, and neuropsychiatric phenotype [7]. Long-COVID is mainly observed in the chronically ill and patients with more than five symptoms during the acute phase of the disease. Moreover, the risk of long-COVID will also be higher with increasing age [5,8]. The exact mechanism behind the prolonged symptoms still needs to be identified. Given the broad spectrum of symptoms, it is likely that the mechanism is multifactorial. Proposed mechanisms include endothelial injury and dysfunction, cytokine storm, dysregulation of the immune response, and the ability of coronavirus to evade the immune system. It is important to recognize and identify the symptoms of long-COVID so that the appropriate treatment and follow-up care can be established. The treatment should be all-rounded, including symptomatic treatment, etiological treatment, and psychological support. With the increasing number of SARS-CoV-2 infections, more people are going to be suffering from long-COVID, not to mention the socioeconomic burden it will bring globally. 

## 2. COVID-19 and Musculoskeletal Dysfunction

### 2.1. Osteoarticular Changes in COVID-19 Patients

Although coronaviral infections are mainly linked to respiratory symptoms, skeletal-related risks and complications are also identified. In 2003, SARS-CoV-1 patients presented with osteonecrosis and reduced bone mass, although these symptoms were mainly attributed to the steroid-based therapy [9]. Today, an osteo-metabolic phenotype of COVID-19 has been characterized, including hypocalcemia, chronic hypovitaminosis D, and a high prevalence of bone fragility. This phenotype is associated with a more severe form of the disease and higher mortality [10,11,12]. It was originally categorized as an endocrine/metabolic phenotype, since a link between COVID-19 and endocrine organs and tissues was easily established after observing a higher mortality in patients suffering metabolic diseases, such as diabetes or obesity. The effect on bone and the mineral metabolism was later defined, and subsequently renamed to the osteo-metabolic phenotype. Hypocalcemia was first observed in a thyroidectomized patient [13]. Following this first report, hypocalcemia was reported in 62.6% to 87.2% of COVID-19 patients, depending on the definition criteria [11]. It corresponds with increased markers for inflammation and thrombosis, a higher need for hospitalization, and increased mortality. The low calcium levels are related to acute malnutrition, the actions of the virus, and hypovitaminosis D [11,12]. Additionally, it is hypothesized that a vitamin D deficiency not only influences hypocalcemia, but also poses a higher risk for more severe disease and increased susceptibility to a SARS-CoV-2 infection on its own. In a randomized observational trial, researchers observed that COVID-19 patients had a high prevalence of hypovitaminosis D, and that these patients have a significantly higher mortality risk [14]. The effects of vitamin D are mainly attributed to the immunomodulatory role of vitamin D on the immune system [15,16]. Immobilization due to the bedrest associated with SARS-CoV-2 infection or due to lockdown and quarantine, in combination with vitamin D deficiency and hypocalcemia, can lead to bone demineralization. 

Although the effects of SARS-CoV-2 on bone ageing and demineralization are abundantly investigated, information on joint aging, including cartilage degeneration or synovial inflammation, is currently lacking. 

### 2.2. Musculoskeletal Pain in COVID-19 Patients

Large joint arthralgia in the knee, ankles, shoulder, wrist, and hip was observed in 50% of patients 6 months after SARS-CoV-1 infection. Since no abnormalities could be observed on MRI, it was postulated that the pain was either of neurogenic origin or linked to low-grade synovitis, which could not be observed on MRI. The non-specific joint pain persisted in these patients 2 to 4 years after the initial SARS-CoV-1 infection [9]. Moreover, muscle weakness and myopathy, including myofiber necrosis or atrophy, were also detected in SARS-CoV-1 patients. It was postulated to be immune-mediated, attributed to critical illness, or steroid-induced myopathy [17,18].

Joint pain and myalgia are also common characteristics of COVID-19. The pain develops during the acute phase of the disease and is observed in 25 to 50% of patients. It has been demonstrated that at the onset of SARS-CoV-2 infection, myalgia is associated with a higher chance of developing post-COVID syndrome and the presence of post-COVID musculoskeletal symptoms. In the latter case, the pain of the acute phase does not disappear and becomes an important characteristic of long-COVID [19,20,21,22,23]. Several longitudinal studies conducted in Turkey, France, and Italy followed COVID-19 patients 6 months after discharge. After 6 months, around 60% of patients were still suffering from at least one SARS-CoV-2 related symptom. The most common were fatigue, myalgia, and joint pain with average prevalence of 30%, 20%, and 15% respectively [24,25]. In the majority of patients (65%), joint pain and myalgia were widespread throughout the body. A minority of patients had local joint pain mainly found in the knee, foot-ankle, and shoulder, while local myalgia was observed in the lower leg, arm, and shoulder. Surveys suggest that around 4.6% to 12.1% of patients still suffer from joint pain during the first year after infection [26]. Long-COVID pain has been demonstrated to be associated with fatigue and is more prevalent to occur in women [27].

**Figure 1:**
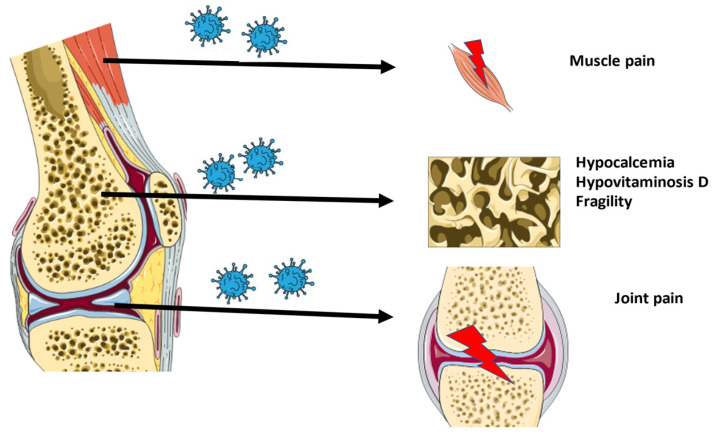
Overview of the musculoskeletal characteristics of long-COVID.

An overview of the musculoskeletal ageing characteristics of long-COVID is shown in Figure 1.

### 2.3. Will COVID-19 Lead to Viral Arthritis?

Several cases of viral arthritis after a SARS-CoV-2 infection have been reported. However, the total number of reported cases compared to the total number of COVID-19 patients is limited, indicating that viral arthritis is a rare complication of the disease. Case studies revealed an early onset of the arthritis days after a severe acute infection, which is cured by non-steroidal anti-inflammatory drug (NSAID) treatment. This complication mostly occurs in males and targets the lower limbs [28,29,30]. It is hypothesized that in severe cases, SARS-CoV-2 induces an auto-immune response causing arthritis. 

In most viral arthritis cases, the virus was not found in the synovial fluid. Recent studies also showed no presence of SARS-CoV-2 in the joint of COVID-19 cadavers after analyzing the synovial fluid, synovial membrane, and bone via real-time polymerase chain reaction [31]. In contrast, one study of a non-hospitalized medium ill patient found traces of nucleic acids of SARS-CoV-2 in the joint [32]. In general, this points to a non-immediate role of SARS-CoV-2 in the onset of musculoskeletal changes after a SARS-CoV-2 infection.

In conclusion, the osteoarticular symptoms and musculoskeletal pain of patients with long-COVID closely resemble early aging characteristics associated with osteoarthritis (OA). These symptoms are probably not connected to viral arthritis, as the prevalence is much higher. The absence of viral RNA in the joint suggests an indirect effect of SARS-CoV-2 on the joint. 

## 3. Early Ageing Alterations in Endothelial and Adipose Tissue of COVID-19 Patients

Recent studies showed that only sporadically nucleic acids of SARS-CoV-2 could be found in the joint [31,32]. Therefore, it seems unlikely that SARS-CoV-2 will directly affect the joint tissue. However, the virus could influence the environment, which can disrupt the normal function of the osteoarticular system. Although knowledge on cartilage and synovium is lacking, bone demineralization and joint pain point to an early OA-like phenotype. It is already known that OA is related to the metabolic syndrome, which combines obesity, hypertension, and diabetes. In this review, we want to explore if SARS-CoV-2 can induce similar changes in adipose and endothelial tissue as the metabolic syndrome, which might lead to the early OA-like phenotype.

### 3.1. Endothelial Dysfunction

The aging vascular system is characterized by endothelial senescence and dysfunction. Aging endothelial cells demonstrate a reduced production of nitric oxide (NO), which is crucial for the control of vascular tone as well as for the regulation of vascular and immunological homeostasis. The reduced availability of NO will impair arterial dilatation in response to biochemical and mechanical stimuli and can be defined as one of the earliest characteristics of hypertension [33]. Moreover, senescent endothelial cells produce an increased level of pro-inflammatory cytokines, which contributes to an unbalanced system of chronic low-grade inflammation [34].

Hypertension and low-grade inflammation will contribute to OA development. Hypertension will reduce the blood flow in the subchondral bone and synovium. This reduces the flow of oxygen and nutrients towards cartilage, which will induce cartilage degeneration. The reduced blood flow also stimulates osteocyte apoptosis and osteoclast function, which affects the subchondral bone function [35,36,37]. Low grade inflammation creates an abundance of pro-inflammatory cytokines, which will contribute to cartilage destruction by inducing the production of matrix metalloproteinases, iNOS, COX-2, and PGE2 [38,39].

SARS-CoV-2 induces endothelial dysfunction via ACE2 either on endothelial cells itself or via neighboring cells. The ACE2 is essential for the entry of SARS-CoV-2 into different types of cells. In healthy individuals, this enzyme is mainly responsible for the conversion of angiotensin II to angiotensin (1–7). The activation of this peptidase, which is a part of the renin-angiotensin system (RAS), results in anti-oxidative, anti-inflammatory, and vasodilative effects. Infection with SARS-CoV-2 leads to a downregulation of ACE2 expression due to endocytosis and the activation of proteolytic cleavage [40]. It is hypothesized that the downregulation of ACE2 in lung endothelial cells, the main target of SARS-CoV-2, leads to inflammation and cytokine storm [41]. Endothelial cells are rich in ACE2, hence, the virus itself can directly cause dysfunction by simply infecting the cells [42]. Additionally, when SARS-CoV-2 infect cells neighboring endothelial cells, an increase in inflammation and cytokine factors can be observed which will, after prolonged exposure, stimulate endothelial dysfunction [43]. 

Endothelial dysfunction plays a pivotal role in the development of COVID-19. It will lead to apoptosis and the loosening of cell-cell interactions between the endothelial cells [43], which will increase the vascular permeability. This will reduce the barrier function and allow the vascular leakage and transportation of the virus to other organs with a high expression of ACE2 resulting in COVID-19 patients with dysfunction of multiple organs. Moreover, endothelial dysfunction will activate the coagulation cascade, which induces thrombus formation. Finally, it will increase inflammation and lead to cytokine storm, which will aggravate the disease [43,44,45].

### 3.2. Adipose Tissue Dysfunction

Adipose tissue dysfunction, characterized by inflammation and senescence, plays an important role in early aging and obesity. Chronic sterile inflammation, a typical phenomenon in aging tissue, also appears in aging adipose tissue. Consequently, the aging adipose tissue demonstrates a changed expression profile of pro-inflammatory and anti-inflammatory markers with enrichment towards the pro-inflammatory cytokines (IL-6, IL-1β and TNFα) and adipokines (leptin) [46]. During aging, senescence cells also emerge, which stimulate senescence-associated secretory phenotype (SASP) production. SASP is responsible for the secretion of other pro-inflammatory factors, growth factors, and soluble receptors which promote inflammation. Senescence is not only observed in adipocytes themselves, but also in other cells in the adipose tissue, such as pre-adipocytes, T-cells, and B-cells [47,48]. This low-grade inflammation and SASP production will impair adipogenesis, recruit immune cells, and lead to insulin resistance. A similar trend is observed in obese patients. The caloric overload will create stress in adipose tissue, leading to the same cascade of reactions as observed during aging, including cellular senescence and low-grade systemic inflammation [38]. 

It is postulated that the increased expression of adipokines and pro-inflammatory cytokines contributes to joint damage and the development of OA. Receptors for the most prominent adipokines, i.e., leptin and adiponectin, are found in a variety of articular cell types, including subchondral osteoblast, chondrocytes, and synoviocytes. This points to a direct role of the adipokines in the joint function. Leptin is considered to have a catabolic function by increasing the expression of matrix metalloproteinases and aggrecanases, while decreasing proteoglycan production. Adiponectin has a dual role, both catabolic and anabolic, depending on its concentration. This adipokine increases the expression of matrix metalloproteinases and degrades aggrecan, while at low concentrations, chondrocytes proliferation and proteoglycan production are observed [35,36,37]. Similarly, the pro-inflammatory cytokines (IL-1 and TNFα) contribute to cartilage destruction by inducing the production of matrix metalloproteinases, iNOS, COX-2, and PGE2 [38,39].

SARS-CoV-2 can induce adipose tissue dysfunction through its influence on ACE2, adiponectin concentration, and apoptosis. SARS-CoV-2 can directly affect adipose tissue, as it is one of the tissues with the highest expression of ACE2. Via ACE2, SARS-CoV-2 can enter the adipose cells, after which they potentially act as viral reservoir resulting in longer viral shedding. Additionally, the viral entry will change the expression of the RAS system in adipose tissue. This system plays an important role in adipose formation. Angiotensin II stimulates lipogenesis and decreases lipolysis in white adipose tissue, leading to adipose hypertrophy and lipid deposition, which is related to inflammation, insulin resistance, and obesity [49,50,51,52]. Moreover, 51% of COVID-19 patients presented with hyperglycemia [53]. It was observed that hyperglycemia was predominantly related to insulin resistance rather than pancreatic beta-cell failure. The study further linked insulin resistance to a decreased concentration of adiponectin, which was shown to be induced by SARS-CoV-2 infection in hamsters [54]. The dysregulation of adipokines, a typical marker for the aging adipose tissue, indicates that SARS-CoV-2 can contribute to adipose tissue dysfunction and the aging process. Finally, after viral entry, the adipose cells can undergo apoptosis or necrosis, which can lead to a meta-inflammation status [55]. 

**Figure 2:**
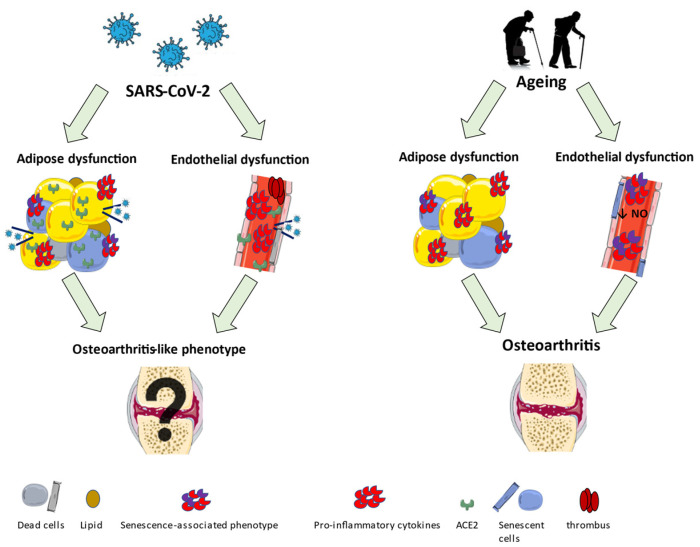
An overview of the early aging alterations in adipose and endothelial tissue due to SARS-CoV-2 infection or ageing.

An overview of the early aging alterations induced by aging or SARS-CoV-2 is shown in Figure 2. 

## 4. Neuronal Sensitization in COVID-19 Patients

Articular pain is an important characteristic of OA and is mainly caused by local pathological changes. Pain-sensing afferent neurons and nociceptors are present in different parts of the joint, including subchondral bone and synovium, in contrast with cartilage, which is aneural. Therefore, bone marrow lesions and synovitis will cause pain, while cartilage damage is not directly linked to pain. The nociceptors will be triggered by both mechanical stimuli and chemical stimuli, including pro-inflammatory factors. However, the level of pain does not always correlate to the radiographic image. This can be attributed to central or peripheral sensitization, in which the threshold for sensing pain is lowered. This leads to hyperalgesia and allodynia [56,57]. 

Pain is an important characteristic of acute as well as long-COVID. Acute pain is unlikely to be related to the direct viral invasion of nociceptors. Presumably, acute pain is associated with the production of cytokines as a response to the infection. Several pro-inflammatory cytokines have been demonstrated to be elevated in bronchial lavage fluid and peripheral blood mononuclear cells in patients with COVID-19, including CCL2/3/4, CXCL2, peptide hormone EREG, IL-1B, IL-6, as well as TNF [58]. The produced cytokines, including IL-1 and IL-6, can interact with nociceptors and induce pain. Acute pain could also originate from the direct viral invasion of muscle cells. Muscle cells have ACE2 receptors that act as an anchoring point for SARS-CoV-2. Viral invasion induces local inflammation and pro-inflammatory cytokines, which result in pain [58].

It could be postulated that long-COVID-related pain is associated with the viral influence on nociceptors in the central and peripheric nervous system. SARS-CoV-2 has been found not only in the peripheral nervous system, but also in the central nervous system, which it enters through direct and indirect pathways. Firstly, it is hypothesized that SARS-CoV-2 can infect the endothelial cells and epithelial cells of the blood-brain barrier, which increases the permeability of the barrier and allows viral entry. Secondly, SARS-CoV-2 may enter the central nervous system via retrograde axonal transport using the peripheral nerve terminals, for example, the terminal olfactory nerve or the enteric nervous system. Finally, viral entry could also be facilitated by infected macrophages and monocyte, which behave as carriers [59,60,61]. It has been demonstrated via mRNA and protein expression that ACE2 is present on the dorsal root ganglia (DRG) in the skin, luminal organs, and meninges. This opens the door for the viral infection of DRG nociceptors, which will disrupt the local RAS and create a disturbance in the balance between the neuromodulation systems of nociception, which can induce pain [62,63]. SARS-CoV-2 infection will decrease the expression of ACE2, which will lead to an accumulation of angiotensin II, and induce pain by binding the angiotensin II type 1 receptor (AT1R), leading to the phosphorylation of p38 mitogen-activated protein kinase, and reduction in angiotensin (1–7). This will decrease PI3K, MAPKs, and JNK pain signaling by attenuating pro and anti-inflammatory cytokine production [64]. The anti-nociceptive role of angiotensin (1–7) has been demonstrated in bone [65]. Besides the direct effect of SARS-CoV-2, the virus can also indirectly induce pain by its excessive immune response, which can lead to sensory cell injury and systemic damage, further contributing to the pain manifestation. SARS-CoV-2 can induce a so-called cytokine storm characterized by a high number of pro-inflammatory markers, which can induce tissue damage and lead to pain [58,63].

**Figure 3:**
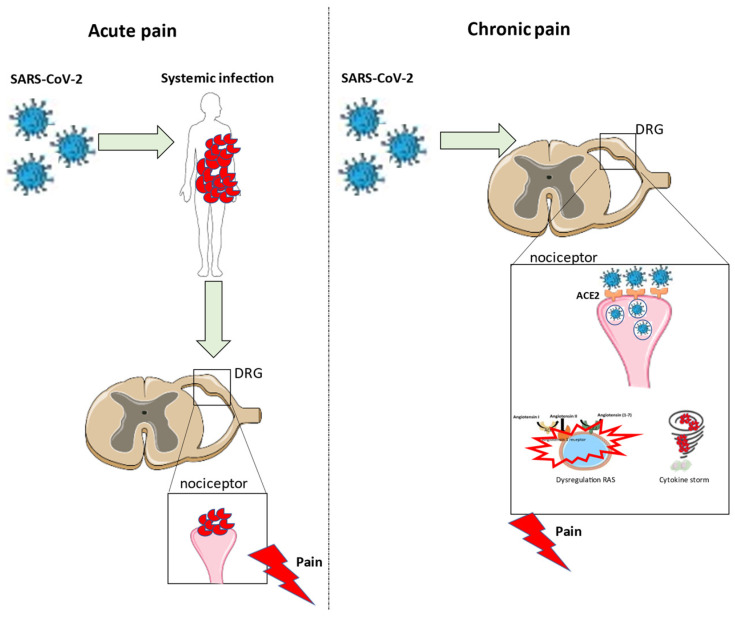
Overview of the development of acute and chronic pain after a SARS-CoV-2 infection.

An overview of pain development due to a SARS-CoV-2 infection is shown in Figure 3.

## 5. Exploring Underlying Molecular Mechanisms

When evaluating the early aging alterations and neuronal sensitization in COVID-19 patients, it is evident that two molecular systems are crucial to accommodate the SARS-CoV-2-induced changes: the RAS and the immune system.

### 5.1. Renin-Angiotensin System

The RAS contains several enzyme (the angiotensin-converting enzyme 1 (ACE1) and ACE2), receptors (AT1R and angiotensin II type 2 receptor (AT2R)), and intermediate products (Angiotensin I, II and angiotensin (1–7)), crucial to maintaining blood pressure and fluid homeostasis in the human body, while dysfunction leads to hypertension [66]. ACE2, an important enzyme of RAS, is known to be the main molecular anchoring point for the viral entry of SARS-CoV-2. The binding of SARS-CoV-2 to ACE2 and the following disturbance in the RAS system has shown to be involved in the initiation of endothelial and adipose dysfunction, and neuronal sensitization [41,42,49,50,51,52,62,63]. As described above, this could potentially induce pain and OA-like changes in the joint and bone.

Moreover, the local expression of the RAS in musculoskeletal tissue has been defined [67]. The importance of the local RAS system in OA has been demonstrated by in vitro administration of angiotensin II to chondrocytes, which reduced chondrocyte viability and metabolism [68]. Moreover, AT1R expression in cartilage is associated with knee OA and plays a role in cartilage degeneration in hypertensive mice [69]. It is hypothesized that OA reflects the depletion of angiotensin (1–7) and stimulates the angiotensin II—AT1R pathway. Additionally, angiotensin-converting enzyme inhibitors (ACEI) and AT1R blockers are associated with improvement of the disease [66]. 

Therefore, it could be postulated that ACE2 and the RAS disturbance forms the molecular machinery underlying long-COVID and its associated OA-like phenotype. 

Modulating the expression of this protein is mostly studied using RAS modulators. Several studies investigated angiotensin receptor blockers (ARB) and angiotensin-converting enzyme inhibitors (ACEI) for their effect on ACE2 expression and activity. Both ARB and ACEI increase the ACE2 expression on the mRNA level, but this could not be detected on protein level. Moreover, ACEI did not change the activity of ACE2, while an increase in activity could be observed after the administration of ARB. Therefore, it can be postulated that ACEI cannot alter the progression of COVID-19, while ARB could improve the symptoms. Several other RAS modulators are proposed as a therapy for COVID-19. This comprises all treatments from stimulating the Angiotensin (1–7)/MAS axis to inhibiting the ACE2/Angiotensin II/AT1R axis. Besides modifying the ACE2 expression and activity, drugs that prevent viral entry via the ACE2 receptor can also have a beneficial effect. This includes soluble recombinant ACE2, which competes with the endogenous ACE2; chloroquine, which glycosylates ACE2 to prevent binding; and camostat mesylate, which is a TMPRSS2 inhibitor [70,71]. 

Interestingly, it has been reported that the viral entry and replication of SARS-CoV-2 depends on the circadian rhythm. The silencing of the major circadian clock transcription activator Bmal1 in lung cancer cells reduced SARS-CoV-2 entry, while overexpression increased entry. This was associated with ACE2 protein expression, which follows a circadian rhythm on the post-transcriptional level. Moreover, the study demonstrated that the circadian clock not only impacts viral entry, but also virus replication in the lung cancer cells [72]. A recent study tested several small molecules known to modify the circadian clock, to inhibit viral replication. They targeted the main protease (3C-like protease), which is responsible for the cleavage of polyproteins into polypeptides required for viral replication. They observed that the circadian clock modulator SRT2183 could inhibit the function of the main protease and may be implemented to block the viral replication of SARS-CoV-2 [73].

### 5.2. COVID-19 and Systemic Inflammation

Systemic inflammation is a typical characteristic of a viral infection and is essential for recovery. However, in severe cases of COVID-19, SARS-CoV-2 can overstimulate the immune response, leading to cytokine storm and T-cell lymphopenia [74]. These phenomena often co-occur, and several hypotheses exist all pointing to highly intertwined underlying mechanisms. One of them postulates that the immune evasion strategy of SARS-CoV-2 can hamper the interferon pathway and shift the cytokine balance towards pro-inflammatory cytokines. As a result, a delayed peak of interferon can be observed that will lead to delayed recruitment of neutrophils and delayed viral clearance, which will prolong the initial response, further increasing the level of cytokines [75]. The prolonged immune response will eventually lead to T-cell exhaustion and lymphopenia. Additionally, the delayed peak of interferon can also reduce T-cell expansion and departure from the lymphoid organs, initiating lymphopenia. However, it could also be hypothesized that the overexpression of cytokines is a response to the reduction of lymphocytes. In the latter case, the virus induces lymphopenia either through the direct viral invasion of lymphocytes via ACE2, causing cell death, or via direct viral invasion of the lymphoid organs, destroying the tissue, which reduces the number of lymphocytes [74,76,77]. Moreover, SARS-CoV-2 also stimulates a variety of T-cells to promote the production of IL-1β and IL-6, and recruit more immune cells to produce cytokines, which can further contribute to the development of the cytokine dysregulation [78]. The systemic inflammation and the overstimulation of the immune response will contribute to endothelial and adipose tissue dysfunction, and neuronal sensitization [43,58,63]. This can be the trigger for OA-like changes in COVID-19 patients. 

Immunomodulatory therapy, including corticosteroids, tocilizumab, and (hydroxy)chloroquine, can modify the immune response to a SARS-CoV-2 infection. Corticosteroids are typically used to control the severity of the infection. However, long-term use and high doses have more adverse effects than useful. Therefore, the usage of corticosteroids should be well controlled. Tocilizumab is an antibody targeting the IL-6 receptor and was originally developed to treat rheumatoid arthritis. Large, randomized multi-center trials are currently conducted to further understand the possible advantages of this therapy. Moreover, other therapies targeting pro-inflammatory cytokines, such as IL-1, TNF, and JAK, are currently being investigated. Chloroquine and hydroxychloroquine are antimalarial drugs which increase the endosomal pH, prevent receptor glycosylation, and could suppress the cytokine storm. Multiple studies investigated the combination of hydroxychloroquine with azithromycin, which could further enhance the effects. Although promising, most studies have multiple limitations questioning the validity of the data. Therefore, the use of hydroxychloroquine for the treatment of COVID-19 remains controversial [79,80,81]. 

Moreover, it is known that several aspects of the human immune system are under control of the circadian clock, including the regulation of inflammatory cytokines, neutrophil maturation, and the differentiation of immune cells, such as Th-cells [82,83]. Thus, the circadian clock could affect the host’s immune response [84]. Based on the peak of the circadian oscillation of the inflammatory markers, the best moment of therapy can be defined. This is called chronotherapy, and it allows the optimization of the efficacy of treatment. This is especially important for anti-inflammatory and immunomodulating therapy [85].

**Figure 4:**
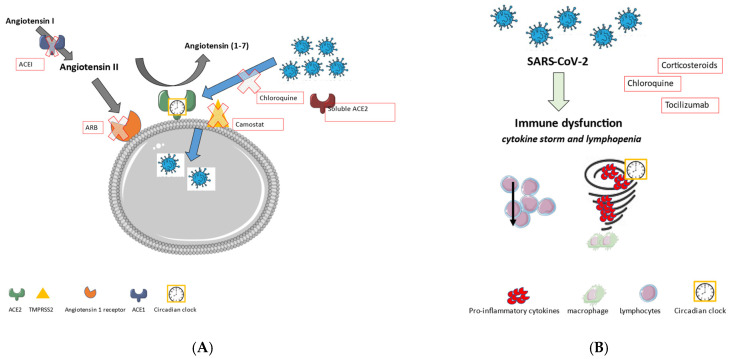
(**A**) The renin-angiotensin system (RAS) and its involvement in SARS-CoV-2 entry into the cells. In the red boxes, the possible treatments of a SARS-CoV-2 infection involving the RAS can be found. (**B**) The immune system and its involvement in a SARS-CoV-2 infection. In the red boxes, the possible treatments of a SARS-CoV-2 infection involving the immune system can be found.

An overview of the RAS and its involvement in SARS-CoV-2 entry into the cells is shown in Figure 4A. Figure 4A also displays possible treatment strategies involving the RAS. Figure 4B shows the role of the immune system and possible treatment options. 

## 6. New Approach in Treating Long-COVID: The Role of the Nicotinic Cholinergic Receptor

Most studies investigating the role of ACE2 show an increased expression in the lungs of smokers or patients with chronic obstructive pulmonary disease (COPD) [86,87,88,89]. This has been attributed to inflammation and the nicotinic cholinergic system. First, smoking and COPD induce inflammation, which can cause an upregulation of ACE2. Secondly, nicotine itself was shown to increase the ACE2 expression in human bronchial epithelial cells. This effect was reduced after the administration of alfa-bungarotoxin, a nicotinic acetylcholine receptor (nAChR) antagonist [90]. Additionally, the gene expression of ACE2 showed a positive correlation with the gene for the nicotinic Alpha 7 subunit of the cholinergic receptor (CHRNA7) (Pearson correlation coefficient of 0.54) [91]. This points further to a direct role of nicotine in the upregulation of ACE2. The higher enzymatic activity of ACE2 after nicotine administration reduces the amount of angiotensin II in favor of Angiotensin (1–7) [92], which will then promote the anti-inflammatory pathways. Moreover, the administration of nicotine could stimulate the cholinergic anti-inflammatory pathway through the alfa7-nAchR and the vagal nerve [93,94]. This points to a role for nicotine or nicotinic receptor agonists in the treatment of inflammatory diseases, including viral infections such as COVID-19.

Additionally, nicotine itself can act as a competitive antagonist for the direct interaction of SARS-CoV-2 with the nAchR. It has been proven that an amino acid sequence in the receptor-binding domain of the spike protein of SARS-CoV-2 is similar to alfa-bungarotoxin and therefore is a good match for the alfa 7 and alfa 9 receptor [95]. The SARS-CoV-2 interaction with the nicotinic receptor will inhibit the nicotinic cholinergic system and dysregulate the Angiotensin II/Angiotensin (1–7) balance in favor of the pro-inflammatory status. The administration of nicotine could counteract this interaction and lower the angiotensin II expression. This further confirms that nicotine could form an interesting therapy to treat COVID-19. 

It needs to be noticed that the dominant negative duplicate CHRFAM7A, the human partial duplication of the CHRNA7 gene which encodes for the α7nAchR, is decreased in patients with a more severe form of COVID-19. A reduced CHRFAM7A expression increases the ion channel function and stimulates the anti-inflammatory effect [96]. This needs to be taken into account when considering treatment strategies, as it has proven to interfere with nicotine-based therapy in the past [97]. In contrast, some studies also estimated a potential influence of nicotine on the development of COVID-19 [98]. More studies are required to further clarify its exact role. 

The nicotinic cholinergic system also plays an important role in joint pain and dysfunction, as observed in the OA pathology [99]. The classical cholinergic anti-inflammatory pathway was recently evaluated to reduce OA-related inflammation [100]. Additionally, the cholinergic system influences the different substructures of the joint attenuating the OA-related dysfunction. The cholinergic system stimulates chondrocyte proliferation, early mineralization, and delays differentiation. It restores the subchondral bone structure through osteoblast proliferation and osteoclast apoptosis [99]. Therefore, attenuating COVID-19 through the nicotinic cholinergic system could also improve its associated OA-like phenotype. 

**Figure 5:**
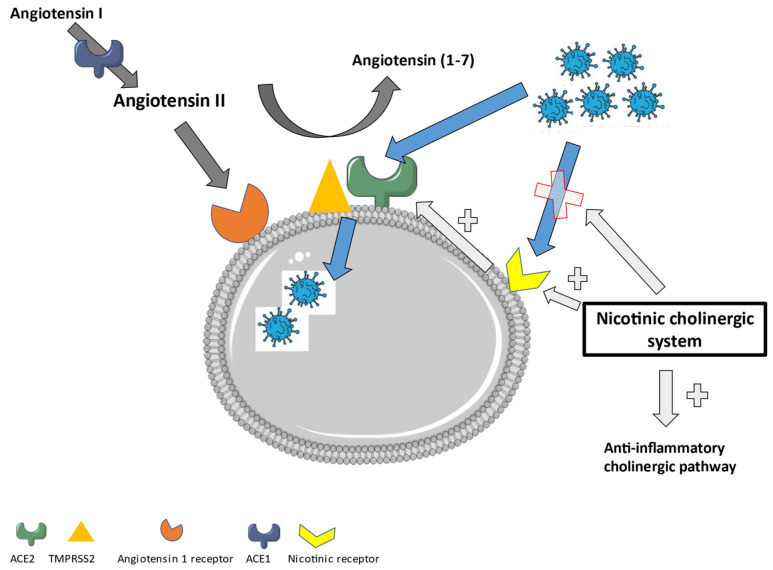
The role of the nicotinic cholinergic system in a SARS-CoV-2 infection.

In Figure 5, an overview of the role of the nicotinic cholinergic system in a SARS-CoV-2 infection is displayed. 

## 7. Conclusions and Hypothesis

Patients suffering from COVID-19 displayed hypocalcemia, vitamin D deficiency, and are often immobilized due to the disease, which will contribute to bone demineralization. Moreover, the patients regularly present with joint and muscle pain. These symptoms resemble early aging characteristics observed during the development of OA. Although no viral presence in the joint could be observed, early aging environmental changes could contribute to the development of this early OA-like phenotype.

Subsequently, the impact of SARS-CoV-2 on the initiation of endothelial and adipose tissue dysfunction was analyzed and compared to early OA. The same concept was applied to analyze neuronal sensitization in COVID-19 and OA. A large similarity between COVID-19 and OA patients was observed regarding endothelial and adipose tissue dysfunction and neuronal sensitization. The SARS-CoV-2 induced changes were mainly attributed to the expression of ACE2 on the respective cells, the disruption of the (local) RAS, and inflammation. Moreover, disruption of the RAS itself could also give rise to musculoskeletal problems.

Finally, drugs that target the renin-angiotensin and immune system were explored as a treatment option for COVID-related musculoskeletal alterations. This includes RAS modulating therapy, circadian clock modulators, immunomodulators, and chronotherapy. Attention was also given to a new approach to treat long-COVID, i.e., the nicotinic cholinergic system. 

This review wants to pave the road for a new aging paradigm in long-COVID. We believe that SARS-CoV-2 contributes to early aging perturbations, such as endothelial and adipose tissue dysfunction, and we attempt to explain the most frequent long-lasting symptoms of SARS-CoV-2 infection as the result of this accelerated aging process.

## Abbreviations

SARS-CoV-1	Severe Acute Respiratory Syndrome Coronavirus-1
MERS-CoV	Middle East Respiratory Syndrome Coronavirus
SARS-CoV-2	Severe Acute Respiratory Syndrome Coronavirus-2
COVID-19	Coronavirus disease emerged in 2019
WHO	World Health Organization
ACE2	Angiotensin-Converting enzyme 2
MRI	Magnetic Resonance Imaging
NSAID	Non-steroidal anti-inflammatory drug
NO	Nitric Oxide
OA	Osteoarthritis
iNOS	Inducible nitric oxide synthase
COX-2	Cyclooxygenase 2
PGE2	Prostaglandin E2
RAS	Renin-angiotensin system
IL-6	Interleukin 6
IL-1β	Interleukin 1 beta
TNFα	Tumor necrosis factor alfa
SASP	Senescence-associated secretory phenotype
CCL2/3/4	C–C motif chemokine ligand 2/3/4
CXCL2	Chemokine (C-X-C motif) ligand 2
Peptide hormone EREG	Protein encoded by EREG gene, epiregulin
DRG	Dorsal root ganglia
AT1R	Angiotensin II type 1 receptor
PI3K	Phosphoinositide 3-kinase
MAPKs	Mitogen-activated protein kinase
JNK	c-Jun N-terminal kinases
ARB	Angiotensin receptor blocker
AT1R blocker	Angiotensin II type I receptor blocker
AT2R	Angiotensin II type 2 receptor
ACEI	Angiotensin-converting enzyme inhibitor
TMPRSS2	Transmembrane serine protease 2
Bmal1	Brain and muscle Arnt-like protein-1
Angiotensin (1–7)/MAS axis	Angiotensin (1–7)/ mitochondrial assembly receptor axis
COPD	Chronic obstructive pulmonary disease
nAchR	Nicotinic acetylcholine receptor
CHRNA7	Gene for the Cholinergic Receptor Nicotinic Alpha 7 Subunit
α7nAchR	Alfa7 nicotinic acetylcholine receptor
CHRFAM7A	human partial duplication of the CHRNA7 gene which encodes for the α7nAchR
